# Associations between epigenetic aging and childhood peer victimization, depression, and suicidal ideation in adolescence and adulthood: A study of two population-based samples

**DOI:** 10.3389/fcell.2022.1051556

**Published:** 2023-01-12

**Authors:** L. C. Perret, M-C. Geoffroy, E. Barr, F. Parnet, N. Provencal, M. Boivin, K. J. O’Donnell, M. Suderman, C. Power, G. Turecki, I. Ouellet-Morin

**Affiliations:** ^1^ Department of Psychiatry, McGill University, Montreal, QC, Canada; ^2^ Department of Educational and Counselling Psychology, McGill University, Montreal, QC, Canada; ^3^ Faculty of Health Sciences, Simon Fraser University, Burnaby, BC, Canada; ^4^ School of Criminology, Research Center of the Montreal Mental Health University Institute, Université de Montréal, Montreal, QC, Canada; ^5^ School of Psychology, Université Laval, Québec City, QC, Canada; ^6^ Yale Child Study Center, Yale School of Medicine, New Haven, CT, United States; ^7^ Department of Obstetrics, Gynecology, and Reproductive Sciences, Yale School of Medicine, New Haven, CT, United States; ^8^ Child and Brain Development Program, CIFAR, Toronto, ON, Canada; ^9^ MRC Integrative Epidemiology Unit, Bristol Medical School, Bristol Population Health Science Institute, Bristol, United Kingdom; ^10^ UCL Great Ormond Street Institute of Child Health, University College London, London, United Kingdom

**Keywords:** epigenetic aging, DNA methylation, early-life stress, peer victimization, depressive symptoms, suicidal ideation, longitudinal studies, adolescence

## Abstract

**Background:** Prior studies indicate that peer victimization (including bullying) is associated with higher risk for depression and suicidal ideation across the life course. However, molecular mechanisms underlying these associations remain unclear. This two-cohort study proposes to test whether epigenetic aging and pace of aging, as well as a DNA methylation marker of responsive to glucocorticoids, are associated to childhood peer victimization and later depressive symptoms, or suicidal ideation.

**Methods:** Cohort 1: Epigenome-wide DNA methylation (EPIC array) was measured in saliva collected when participants were 10.47 years (standard deviation = 0.35) in a subsample of the Quebec Longitudinal Study of Child Development (QLSCD, *n* = 149 participants), with self-reported peer victimization at 6–8 years, depressive symptoms (mean symptoms, and dichotomized top 30% symptoms) and suicidal ideation at 15–17 years. Cohort 2: Epigenome-wide DNA methylation (EPIC array) was measured in blood collected from participants aged 45.13 years (standard deviation = 0.37) in a subsample of the 1958 British Birth cohort (1958BBC, *n* = 238 participants) with information on mother-reported peer victimization at 7–11 years, self-reported depressive symptoms at 50 years, and suicidal ideation at 45 years. Five epigenetic indices were derived: three indicators of epigenetic aging [Horvath’s pan-tissue (Horvath1), Horvath’s Skin-and-Blood (Horvath2), Pediatric-Buccal-Epigenetic age (PedBE)], pace of aging (DunedinPACE), and stress response reactivity (Epistress).

**Results:** Peer victimization was not associated with the epigenetic indices in either cohort. In the QLSCD, higher PedBE epigenetic aging and a slower pace of aging as measured by DunedinPACE predicted higher depressive symptoms scores. In contrast, neither the Horvath1, or Horvath2 epigenetic age estimates, nor the Epistress score were associated with depressive symptoms in either cohort, and none of the epigenetic indices predicted suicidal ideation.

**Conclusion:** The findings are consistent with epigenome-wide and candidate gene studies suggesting that these epigenetic indices did not relate to peer victimization, challenging the hypothesis that cumulative epigenetic aging indices could translate vulnerability to depressive symptoms and suicidal ideation following peer victimization. Since some indices of epigenetic aging and pace of aging signaled higher risk for depressive symptoms, future studies should pursue this investigation to further evaluate the robustness and generalization of these preliminary findings.

## Introduction

Peer victimization has been defined as intentional “harm caused by peers acting outside the norms of appropriate conduct” (Finkelhor et al., 2012), and includes bullying which is further characterized by a recurrence of these experiences over time and an imbalance of power between the perpetrator(s) and the victim (Gredler and Olweus, 1993). Peer victimization is a known risk factor for a range of mental health problems across the life course (Moore et al., 2017; Arseneault, 2018; Oncioiu et al., 2021; Rijlaarsdam et al., 2021). To illustrate, experiencing peer victimization in childhood has been longitudinally associated with higher risks of depressive symptoms and severe depression in adolescence (Bowes et al., 2015; Geoffroy et al., 2018), which can persist into adulthood (Takizawa et al., 2014; Klomek et al., 2015; Moore et al., 2017). Furthermore, peer victimization has been associated with suicidal ideation and even suicide mortality (Takizawa et al., 2014; Holt et al., 2015; Klomek et al., 2015; Geoffroy et al., 2016; Geoffroy et al., 2022). Although the association between peer victimization, depressive symptoms, and suicidal ideation is empirically supported by multiple longitudinal studies, the underlying biological mechanisms are still poorly understood.

Existing mechanistic studies have followed many research avenues using genetic, neurobiological, endocrinological, and molecular approaches to explain how the adverse experience of peer victimization increases risk for subsequent mental health problems (Vaillancourt et al., 2013). At the forefront of this search to uncover the early roots of mental health disparities are the neurophysiological systems that translate threats (internal and external) into biological responses to stress, such as the Hypothalamic Pituitary Adrenal axis (HPA) and its main glucocorticoid hormone, cortisol (Shonkoff, 2010; Gunnar, 2020). For example, prolonged cortisol secretion, triggered by repeated verbal and physical abuses perpetrated by peers, had been proposed to affect the activity and neurocircuitry of brain regions rich in glucocorticoid and mineralocorticoid receptors over time, and to have deleterious effects on a myriad of neurobiological processes, including the immune and inflammatory systems (Shonkoff, 2010; Heim and Binder, 2012).

The molecular mechanisms by which early adverse experiences such as verbal and physical abuse may become embedded could be through epigenetic modifications (Hertzman, 2013; Lutz et al., 2015; McEwen, 2017; Lang et al., 2020). Epigenetics refers to non-genetic mechanisms that can regulate gene expression in DNA. DNA methylation is one type of epigenetic mechanism characterized by the addition of a methyl group to a cytosine nucleotide, usually one that is paired with guanine (CpG). There is evidence that DNA methylation may be associated with early adversity and perhaps more so with depression and suicide ideation, but few studies with inconsistent findings have investigated its association with peer victimization. We further describe these gaps and how the present study will explore them.

Firstly, epigenetic mechanisms, especially DNA methylation, have been extensively studied in relation to early adversity, such as perinatal stress and childhood maltreatment (Bick et al., 2012; Szyf, 2013; Suderman et al., 2014; Turecki and Meaney, 2016; Cecil et al., 2020; Provenzi et al., 2020; Parade et al., 2021). However, much less is known about the association between DNA methylation and peer victimization. One study found increased methylation of the serotonin transporter (*5-HTT*) gene in bullied twins (Ouellet-Morin et al., 2013), another study by Efstathopoulos et al. (2018) found higher methylation in the glucocorticoid receptor (*NR3C1*) gene. However, these early studies targeted single genes. In the largest analysis carried out to date, Marzi et al. (2018) tested associations of peer victimization with DNA methylation in twin pairs (*N* = 2,232) at CpG sites located across the genome and within candidate genes, including *5-HTT* and *NR3C1* genes. No DNA methylation differences between victimized children or adolescents and their non-victimized counterparts were uncovered (Marzi et al., 2018). Another two-cohort study (*N* = 1,352) investigated changes in DNA methylation across the genome at two timepoints; 6 and 10 years in the first cohort, and 7.5 and 17 years in the second cohort (Mulder et al., 2020). A significant difference was uncovered at only one CpG site.

This study proposes a complementary approach to previous candidate gene and genome-wide studies by investigating DNA methylation indices, encompassing DNA methylation levels at multiple CpG sites, thought to be relevant to either the experience of peer victimization, or depressive symptoms, or suicidal ideation. As others have done before in the context of adverse childhood experiences, we argue that cumulative indices grouping specific CpG sites known to be responsive to glucocorticoids, or known to indicate the “wear and tear” of aging, or the accelerated pace of aging may help uncover stronger and more robust epigenetic effects of peer victimization. Importantly, we could also use these cumulative indices to test if DNA methylation partly mediates the association between peer victimization and depressive symptoms or suicidal ideation. The use of cumulative indices may overcome some limitations to prior approaches. Namely, epigenome-wide approaches can be limited by the fact that individual CpG sites with small effects may not reach epigenome-wide significance, or may be hard to replicate. At the opposite end of the spectrum, although candidate gene approaches have greater statistical power to detect associations, they may only provide partial evidence when a collection of genes may be involved.

Cumulative epigenetic indicators of biological aging, often referred to as epigenetic age or clocks, have been created to capture the accumulation of changes in DNA methylation linked to aging (Ryan, 2021). One of the most widely known epigenetic age is Horvath’s pan-tissue clock (Horvath1) which is known to strongly correlate with chronological age and was derived from an array of tissues, including blood and saliva (Horvath, 2013). The Horvath1 has been associated with adverse childhood experiences, including abuse and exposure to violence, in samples composed of children and adolescent populations (Sumner et al., 2019), as well as with childhood trauma in adults (Jansen et al., 2021). Notably, only one study has examined whether peer victimization between 0–14 years predicted differences in Horvath1 at 17 years, but no association was found (Tang et al., 2020). More recent indicators of epigenetic aging, such as the Horvath’s Skin-and-Blood clock (Horvath2) (Horvath et al., 2018) and the Pediatric-Buccal-Epigenetic age clock (PedBE) have shown to better predict chronological age using DNA methylation data derived from skin, blood, and buccal epithelial cells (McEwen et al., 2020). In addition to epigenetic age, which is trained to predict chronological age, epigenetic pace of aging has been derived to predict how fast epigenetic aging is occurring. Specifically, the Dunedin Pace of Aging Calculated from the Epigenome (DunedinPACE) was derived based on blood samples from 26–45 year-old participants (Belsky et al., 2022), and its predecessor, the Dunedin Pace of Aging methylation (DunedinPoAm) was based on smaller age range of 28–38 year-old participants (Belsky et al., 2020). Both indices have been linked to adverse childhood experiences, including poverty (Raffington et al., 2021; Belsky et al., 2022; McCrory et al., 2022) and polyvictimization; e.g., child abuse and neglect, peer victimization (Bourassa et al., 2021). However, no study has yet tested if specific types of adverse childhood experiences, such as peer victimization, predict the pace of aging. Finally, other epigenetic biomarkers build on surrogate measures of sensitivity to chemical or hormonal exposures, including glucocorticoids. Notably, Provençal et al. (2020) have created a score (the Epistress) that includes 24 CpG sites that are sensitive to glucocorticoids. Since peer victimization has been repeatedly associated to both the salivary cortisol stress response and cumulative hair cortisol levels (Ouellet-Morin et al., 2011; Ouellet-Morin et al., 2021), the Epistress score represents another cumulative epigenetic index of interest. However, to date, the Epistress score has never been examined in relation to peer victimization. The present study aims to fill this gap in knowledge.

A few studies have nevertheless explored associations between epigenetic age with depressive symptoms and suicidal ideation. Specifically, Horvath1 was associated with depressive symptoms in childhood (Sumner et al., 2019; Tollenaar et al., 2021) and in adulthood in some studies (Whalley et al., 2017; Han et al., 2018), but not all (Beydoun et al., 2019; Oblak et al., 2021; Klopack et al., 2022). To our knowledge, Horvath2 has not been examined with depressive symptoms. Only a few studies have investigated its association with internalizing symptoms (including depression and/or anxiety) with the PedBE. One study has found higher PedBE age among children with internalizing disorder (depression and/or anxiety disorders) (Dammering et al., 2021), although two others reported non-significant findings in similar phenotypes during childhood (Manczak et al., 2021; McGill et al., 2022). In regards to suicidal ideation, only one study, conducted in a clinical sample of patients with schizophrenia, did not find an association with Horvath1 (Dada et al., 2020). Finally, neither the DunedinPACE nor the Epistress score have been examined in relation to depressive symptoms or suicidal ideation. However, DunedinPACE’s predecessor, the DunedinPoAm, had been previously linked to concurrent depression in adults aged between 50 and 87 years (McCrory et al., 2022). In sum, existing evidence on whether cumulative epigenetic indices, such as epigenetic indicators of aging, pace of aging, or the Epistress score, points to some inconsistent association with peer victimization, depressive symptoms, or suicidal ideation, but this investigation remains new. To the best of our knowledge none have formally tested whether these indices partly mediate the associations between peer victimization and depressive symptoms, or suicidal ideation. However, many theoretical frameworks propose such a causal pathway (Heim and Binder, 2012; Vaillancourt et al., 2013).

The main objective of this study was to test longitudinal associations between childhood peer victimization, depressive symptoms, and suicidal ideation in adolescence and adulthood, with cumulative epigenetic indices measured in childhood and adulthood, using the Quebec Longitudinal Study of Child Development (QLSCD) cohort and the 1958 British Birth (1958BBC) cohort. The Horvath2 and PedBE were specifically selected because the QLSCD included DNA derived from saliva samples, whereas the 1958BBC had blood-derived DNA. Using two different cohorts with different tissues measured at distinct developmental periods (10 years, vs. 45 years) may help to unravel robust signals detected across both tissues and age, or potentially identify distinct mechanisms. This study specifically tests whether: 1) childhood peer victimization was associated with epigenetic aging (Horvath1, Horvath2, & PedBE), accelerated pace of aging (DunedinPACE) and the Epistress score; 2) each epigenetic index was associated with depressive symptoms and suicidal ideation measured in adolescence and mid-adulthood; and 3) any of the epigenetic indices partly mediated the association between peer victimization, depressive symptoms, and suicidal ideation.

## Methods

### Sample and procedures

This study uses datasets from two cohorts (Supplemental Figure S1): The QLSCD and the 1958BBC (also known as The National Child Development Study). The QLSCD is an ongoing prospective birth cohort of 2,120 participants born in the Canadian province of Québec in 1997–1998, managed by *Institut de la Statistique du Québec*. Further details about the cohort can be found online (https://www.jesuisjeserai.stat.gouv.qc.ca/) and in the cohort profile (Orri et al., 2021). The Ethics Committee of the *Institut de la Statistique du Québec,* approved each phase of the study, and informed consent was obtained for all participants. A subsample of QLSCD participants provided saliva samples at 10 years of age which were collected by a registered nurse during a home visit, and frozen in secured laboratory freezers for storage until DNA extraction was performed. The QLSCD study sample and analyses included 149 individuals DNA samples and data on peer victimization at 6–8 years, depressive symptoms, and suicidal ideation at 15–17 years (see DNA methylation indices for more information on participant number). No specific inclusion criteria were applied for selecting the samples, other than the availability of variables of interest.

The 1958BBC is an ongoing longitudinal birth cohort including an initial sample of 17,416 participants born in England, Scotland, and Wales during 1 week in March 1958. The final sample of 18,558 participants also included immigrants born in the same week but added in subsequent follow-ups in childhood and adolescence. The cohort profile (Power and Elliott, 2006) and the Centre for Longitudinal Studies at the University College London website contain more information (https://www.cls.ioe.ac.uk). Ethical approval for the 45-year survey was given by the South East Multi-Centre Research Ethics Committee (ref. 01/01/44) and consent was obtained for all participants. A subsample of 1958BBC participants took part in the biomedical sweep, which included a home interview by a research nurse, questionnaires, and blood collection. Blood samples were frozen securely at the clinical study sites samples until DNA extraction was performed. The current study included 238 individuals from the 1958BBC with information on DNA methylation at 45 years, peer victimization at 7–11 years, and suicidal ideation at 45 years. Study analyses with depressive symptoms scores at 50 years in the 1958BBC included 226 participants due to missing data on depressive symptoms scores for 12 participants. No specific inclusion criteria were applied for selecting the samples, other than the availability of variables of interest.

There were no significant differences on key sociodemographic variables between participants included in our analyses and the remainder of each respective cohort. However, the QLSCD study participants who provided DNA methylation data at age 10 years reported lower depressive symptoms later in adolescence compared to non-included participants. Similarly, the participants included in our analyses from both cohorts reported less suicidal ideation compared to those who were not included.

### Description of measures

#### DNA methylation indices

QLSCD: 365 saliva samples at 10 years were collected using the Oragene DNA sample collection kit (DNA Genotek). DNA was extracted and used for genotyping purposes, which reduced the remaining DNA available for DNA methylation quantification. 154 DNA samples were selected for quantification using absorbance (Nanodrop) and fluorometry (Pico green). Bisulfite-conversion was performed with the EZ-96 DNA Methylation Kit (Zymo Research, cat. No. D5004). After excluding 2 missing, 1 duplicate, and 1 poor quality DNA samples, 150 DNA samples were run on the Infinium Methylation EPIC BeadChip Array (Illumina); a methylation array that allows for quantitative interrogation of over 850,000 CpG methylation sites across the genome. Beta values, ranging from 0 to 1, were extracted, processed, and normalized using R software (version 3.6.2). Noob normalization was performed on all samples to calculate the epigenetic aging indicators (described below) following quality control steps. Functional normalization with the first 9 principal components of our control matrix was performed without background correction for the Epistress score. Variance introduced into our data by the array (Beadchip position/batch, etc.) following functional normalization (used for Epistress scores only) was removed with ComBAT using the R package sva. All cross-hybridizing and cross-reactive probes were removed. Probes with a detection *p*-value greater than 0.001 in 25% of samples were removed (cut-offs according to detection of Y chromosome probes in female samples). Cell-type proportions in saliva were estimated as they may confound associations between DNA methylation and exposure to adverse environments or diseases (Middleton et al., 2022). We used the EpiDISH method to estimate cell-type proportions (Epithelial cells, Fibroblasts, B cells, Natural Killer cells, CD4 T cells, CD8 T cells, Monocytes, Neutrophils, Eosinophils) in DNA methylation data derived from saliva samples because it reports robust and reduced noise level compared to the Houseman method, which is more commonly used for blood samples (Teschendorff et al., 2017). A principal component analysis was then performed on all cell types and the resulting two principal components were subsequently statistically controlled for in all epigenetic indices through residuals. One participant was excluded from further analyses because they had very high unadjusted epigenetic aging indicators: Horvath2 and Horvath1 age >10 standard deviations (SD) above the mean. The final sample thus included 149 participants.

1958BBC: The biomedical follow-up at 45 years included 9,426 participants who provided blood samples. Genomic DNA samples were collected from blood in 238 participants at age 45. DNA methylation was measured using the Infinium Methylation EPIC BeadChip Array. Quality of raw microarray data was assessed using standard procedures for detecting outliers, dye bias, signal noise and technical artifacts. After preprocessing (subtracting background signal and removing dye bias), the DNA methylation data was normalized using functional normalization (Fortin et al., 2014) to subsequently calculate the Epistress score (described below). Noob normalization was performed prior to calculating the epigenetic aging and pace of aging indicators (described below). Cell-type proportions (B cells, CD4 T cells, CD8 T cells, Eosinophils, Monocytes, Neutrophils, Natural Killer cells) were estimated with the Houseman method (Houseman et al., 2012). A principal component analysis was then conducted on all cell types, and the resulting three principal components were subsequently statistically controlled for in all epigenetic indices through residuals.

#### Epigenetic aging and DunedinPACE

We used R software (version 4.1.0) to calculate epigenetic aging indicators for both cohorts. Horvath1 utilizes 353 CpGs selected through elastic net regression in samples aged 0–100 (Horvath, 2013). Horvath2 includes 391 CpGs (60 sites overlapping with Horvath1) selected through elastic net regression with greater tissue diversity in samples aged 0–90 years (Horvath et al., 2018). PedBE with 94 CpGs selected through elastic net regression (1 site overlapping with the Horvath1 and Horvath2, and 11 more with the Horvath2), was optimized for pediatric cohorts (0–20 years) (McEwen et al., 2020). The Horvath2 was a key benchmark for comparison because it performs well in both saliva and blood samples. The PedBE was of interest because we tested associations with childhood experiences, and especially in DNA collected at 10 years in the QLSCD cohort. Epigenetic aging indicators (unadjusted scores) were derived using R codes supplied by the authors for each clock in each cohort separately. The DunedinPACE was computed from DNA methylation data using the publicly available scripts (https://github.com/danbelsky/DunedinPACE).

#### Epistress

The Epistress score was derived from DNA methylation data as previously described by Provençal et al. (2020). Briefly, a set of 496 CpG were identified as cross-tissue glucocorticoid sensitive sites following exposure of hippocampal stem cells and whole blood with a synthetic glucocorticoid (dexamethasone). A further subset (24 CpGs) were selected using an elastic net regression to create the Epistress score with corresponding weights reflecting the magnitude of change in DNA methylation following dexamethasone treatment (Provençal et al., 2020). One CpG of the original 24 CpGs included in the Epistress score did not pass quality control tests and was thus substituted by the next most sensitive CpG. The weighted Epistress score was derived for each cohort in R.

#### Epigenetic index residuals

Residuals were calculated for each epigenetic index to account for potential confounders.

QLSCD: The residual scores of each epigenetic index included 1) decimal age in years at the 10-year follow-up, 2) principal components of cell heterogeneity, and 3) the most correlated principal components associated to genetic ancestry with epigenetics indices (i.e., the first three), and 4) Body Mass Index (BMI) at 10 years. PedBE and Horvath2 unadjusted scores for one participant were winsorized (scores beyond 3 SD and replaced by the closest score within the ±3 SD threshold). All residual scores were standardized into z-scores.

1958BBC: The residual scores of each epigenetic index included 1) decimal age in years at the 45-year follow-up, 2) principal components of cell heterogeneity, 3) smoking status at 42 years (0 = never, 1 = occasional or ex smoker, 2 = current smoker), and 4) BMI at 45 years. PedBE and Epistress unadjusted scores were winsorized for five participants. All residual scores were standardized by calculating z-scores.

#### Peer victimization

QLSCD*:* Peer victimization was self-reported at 6, 7, and 8 years using a 7-item modified version of the Self-reported Peer Victimization Scale (Ladd and Kochenderfer-Ladd, 2002). Participants reported how often (0 = never, 1 = once or twice, 2 = more often) they experienced physical (i.e., “pushed, hit or kicked”), verbal (i.e., “called names, insulted, said mean things to you,” “teased you in a mean way”), relational peer victimization (i.e., “did not let you play with or be part of his or her group,” “said bad things about you to other children”), and property attacks (i.e., “took away things that belong to you without asking your permission and without giving them back to you”, “purposely broke something that is yours”) since the beginning of the school year. Cronbach’s alpha was 0.73 at 6 years, 0.76 at 7 years, and 0.74 at 8 years. Peer victimization at 6–8 years was calculated as the mean score at each time point and then averaged. Peer victimization between each time point were moderately correlated (*r*s range from .26 to .51, *p* < .001).

1958BBC*:* Peer victimization was reported by mothers when participants were aged 7 and 11 years. At each time point, mothers were asked if their child was “bullied by other children” (0 = never, 1 = sometimes, or 2 = frequently). Peer victimization at 7–11 years was calculated as the mean score. Peer victimization at 7 and 11 years were moderately correlated (*r* = 0.32 *p* < .001).

Childhood peer victimization scores were standardized into z-scores in each cohort.

#### Depressive symptoms

QLSCD: Depressive symptoms in the past year were self-reported at 15 and 17 years using eight items from the Mental Health and Social Inadaptation Assessment (MIA) (Côté et al., 2017; Geoffroy et al., 2018). The MIA is not a diagnositic tool, but its items reflect symptoms in the Diagnostic and Statistical Manual of Mental Disorders, 5th edition. Participants reported how often (0 = never, 1 = sometimes, 2 = often) they experienced each symptom in the past 12 months; “Nothing was fun for me, I was not interested in anything,” “I felt sad and unhappy,” “I lacked energy or felt tired,” “I lost interest in things I usually like,” “I felt I could not do anything well,” “I felt I was not as good-looking or as smart as other people,” “Doing even little things made me feel really tired,” “I had trouble thinking clearly.” Cronbach’s alpha was .84 at 15 years and 0.74 at 17 years. Pearson correlations between ages were *r* = .495 (*p* < .001) between 15 and 17 years.

1958BBC: Depressive symptoms in the past 4 weeks were self-reported at 50 years using the emotional wellbeing scale of the SF-36 (also known as the Mental Health Index- five items) (Ware, 2000; Taft et al., 2001). Participants rated five items on a 6-point scale ranging from “all” to “none of the time”; “Have you been a very nervous person?,” “Has felt so down in the dumps nothing could cheer you up,” “Have you felt downhearted and low?,” “Have you felt calm and cheerful?,” “Have you been a happy person?.” Cronbach’s alpha was 0.87 at 50 years.

In both samples, responses were summed to produce a continuous score of depressive symptoms, which was standardized into Z-scores to ease interpretation. In addition, to explore if these cumulative epigenetic indices better predicted the occurrence of more severe symptoms, rather than according to its continuous distribution, participants with elevated depressive symptoms scores in the top 30% were identified. This also facilitated comparison with previous studies that focused on elevated depressive symptoms.

#### Suicidal ideation

QLSCD: Suicidal ideation frequency was measured by one item; “In the past 12 months, did you ever think about suicide?” and scored 0 = never, 1 = rarely, 2 = fairly often, 3 = very often, at 15 or 17 years (13 years data was used for 10 participants with missing information at either age). Participants who indicated having suicidal ideation “rarely” to “very often,” at either 15 or 17 years, were coded as “yes,” while those who answered never at both ages were coded as “no”.

1958BBC: Suicidal ideation was measured using the depressive ideas subscale of the Clinical Interview Schedule-Revised (Lewis et al., 1992). The depressive ideas subscale has previously been used and found to associate with childhood adversity (Stansfeld et al., 2017). The subscale evaluates five symptoms in the past 7 days by summing affirmative answers (0 = no, 1 = yes) to the following questions: “Have you on at least one occasion felt guilty or blamed yourself when it has not been your fault?,” “During the past week have you been feeling you are not as good as other people?,” “During the past week have you felt hopeless about your future?”; “In the past week have you felt that life is not worth living?,” “In the past week have you thought of killing yourself?.” This five-point scale was dichotomized (⩾2 = suicidal ideation) to indicate a clinically significant symptoms based on the CIS-R scoring protocols (Lewis et al., 1992).

#### Covariables

QLSCD: Family socioeconomic status at 5 months was an aggregate of annual gross income, parental education level, and occupational prestige, standardized into z-scores (Willms and Shields, 1996). Sex and birthweight (grams) information were obtained from medical records. Height and weight were collected at 10 years, and BMI was derived from these variables.

1958BBC*:* Socioeconomic status was based on the father’s occupation at birth (imputed at 7 years if missing) using the Registrar General’s Social classification, grouped as I or II (professional/managerial), III-NM (skilled non-manual), III-M (skilled manual) and IV and V (semi-skilled and unskilled manual, including single mother households). Information on sex and birthweight (ounces) was collected at birth from medical records. Seven participants had missing information on birthweight (2.9% of the sample). We replaced these missing values with the mean birthweight of participants with the same gestational age (in days). BMI was obtained at 45 years using weights and heights measured by a nurse.

### Statistical analyses

First, we examined associations between peer victimization (QLSCD: 6–8 years; 1958BBC: 7–11 years) and depressive symptoms (QLSCD: 15–17 years; 1958BBC: 50 years), or suicidal ideation (QLSCD: 15–17 years; 1958BBC: 45 years). Second, we tested associations between the epigenetic indices (residuals) and peer victimization, depressive symptoms, or suicidal ideation in a series of linear and logistic regressions according to the continuous and dichotomous variables. In each case, two regression models were conducted: Model one was unadjusted, whereas Model two adjusted for sex, socioeconomic status, and birthweight. To avoid unnecessary tests and thus minimize the likelihood of identifying false positives, the mediation analyses were only conducted for epigenetic indices with significant associations with both peer victimization and depressive symptoms or suicidal ideation. All the above-mentioned analyses we conducted using IBM SPSS Statistics 23.

## Results

### Descriptive of the epigenetic indices

Table 1 describes the unadjusted scores of each epigenetic aging, pace of aging and the Epistress score. In the QLSCD, the mean chronological age was 10.47 years (SD ± 0.35, range = 9.7–11.3 years). The unadjusted Horvath1 and PedBE epigenetic age accurately estimated the chronological age within the appropriate range (9.83 years (SD ± 1.49), 9.70 years (SD ± 0.78) and respectively). The Horvath2 slightly underestimated chronological age to an average of 7.04 years (SD ± 0.82). The mean age in the 1958BBC was 45.13 years (SD ± 0.37, range = 44.53–45.93 years). Horvath1 epigenetic mean age was 41.72(SD ± 4.86), which was close to the mean chronological age. While the Horvath2 overestimated the mean chronological age (59.16 years, SD ± 3.07) by approximatively 14 years, the PedBE greatly underestimated chronological age by about 35 years (9.91 years (SD ± 0.94)).

**TABLE 1:** Descriptive statistics of chronological and epigenetic age estimates, DunedinPACE, and Epistress in both cohorts^a^.

QLSCD	Chronological age	Horvath1	Horvath2	PedBE	DunedinPACE	Epistress
Mean (±SD)	10.47 (0.35)	9.83 (1.49)	7.04 (0.82)	9.70 (0.78)	1.17 (0.16)	−1.02 (0.22)
Range (min to max.)	9.7 to 11.3	6.54 to 14.53	5.06 to 9.71	8.25 to 12.09	0.82 to 1.51	−1.58 to −0.58

1958BBC		Horvath1	Horvath2	PedBE	DunedinPACE	Epistress
Mean (±SD)	45.13 (0.37)	41.72 (4.86)	59.16 (3.07)	9.91 (0.94)	1.00 (0.12)	−1.16 (0.28)
Range (min to max.)	44.53 to 45.93	28.49 to 55.19	50.94 to 67.25	7.21 to 12.52	0.71 to 1.39	−1.80 to −0.50

^a^ Unadjusted winsorized epigenetic clocks, DunedinPACE, and Epistress.

QLSCD, Data were compiled from the final master file (1998–2015), © Gouvernement du Québec, Institut de la Statistique du Québec.

Max *N* based on data available for epigenetic clocks and Epistress (QLSCD: *n* = 149; 1958BBC: *n* = 238).

Table 2 presents the correlations between the three epigenetic indicators of aging, pace of aging (DunedinPACE) and the Epistress score. In the QLSCD, the Horvath1 was correlated with the other two epigenetic age estimates, Horvath2 and PedBE (*r* = .310, *p* < .001; *r* = .271, *p* < .001, respectively). The PedBE was also correlated to the Horvath2 (*r* = .291, *p* < .001). The DunedinPACE was correlated to Horvath1 and the Epistress score (*r* = −.175, *p* = .032; *r* = −.220, *p* = .007, respectively). Lastly, the Epistress inversely correlated with Horvath2 and PedBE (*r* = −.165, *p* = .045; *r* = −.178, *p* = .030; *r* = −.220, *p* = .007, respectively), but was not significantly correlated with Horvath1. In the 1958BBC, Horvath1 correlated with Horvath2 (*r* = .415, *p* < .001). The PedBE only correlated with the Horvath2 (*r* = .247, *p* < .001). The Epistress score only correlated with Horvath1 (*r* = .165, *p* = .011). DunedinPACE did not correlate with any of the other epigenetic indices. These patterns of correlation indicate that while some overlaps exist between the cumulative epigenetic indices, due in part to common CpG sites, they also captured distinct variance, hence pointing to their complementary value. Since the PedBE grossly underestimated chronological age, that the expected correlation with Horvath1 was not present, that this epigenetic aging indicator has not been optimized for use in DNA extracted from blood samples and was initially derived for youth aged 0–20 years, we decided to exclude this index from further analyses in the 1958BBC.

**TABLE 2:** Correlation matrix for the epigenetic age estimates, DunedinPACE, and Epistress in both cohorts

QLSCD		Horvath1	Horvath2	PedBE	DunedinPACE	Epistress
Horvath1	Pearson *r*	—				
	*p*-value					
Horvath2	Pearson *r*	**0.310**	—			
	*p*-value	**<0.001**				
PedBE	Pearson *r*	**0.271**	**0.291**	—		
	*p*-value	**<0.001**	**<0.001**			
DunedinPACE	Pearson *r*	−**0.175**	0.054	−0.095	—	
	*p*-value	**0.032**	0.511	0.251		
Epistress	Pearson *r*	0.032	**-.165**	−**0.178**	−**0.220**	—
	*p*-value	0.701	**0.045**	**0.030**	**0.007**	

1958BBC		Horvath1	Horvath2	PedBE	DunedinPACE	Epistress
Horvath1	Pearson *r*	—				
	*p*-value					
Horvath2	Pearson *r*	**0.415**	—			
	*p*-value	**<0.001**				
PedBE	Pearson *r*	−0.022	**0.247**	—		
	*p*-value	0.736	**<0.001**			
DunedinPACE	Pearson *r*	0.033	0.022	−0.020	—	
	*p*-value	0.611	0.738	0.759		
Epistress	Pearson *r*	**0.165**	0.012	0.114	0.080	—
	*p*-value	**0.011**	0.852	0.081	0.221	

Bold values are significant at *p* < 0.05. Notes: Epigenetic index residuals adjusted for principal components for cell type heterogeneity, smoking status at 42 years (1958BBC only), and body mass index (QLSCD: 10 years; 1958BBC: 45 years), and principal components for ancestry (QLSCD only), and chronological age (QLSCD, decimal age at the 10 years data collection; 1958BBC, decimal age at the 45 years data collection).

Max N based on data available for epigenetic clocks, DunedinPACE, and Epistress. (QLSCD: *n* = 149; 1958BBC: *n* = 238).

QLSCD, Data were compiled from the final master file (1998–2015), © Gouvernement du Québec, Institut de la Statistique du Québec.

### Associations between peer victimization and depressive symptoms

Linear and logistic regression analyses indicated that, in the QLSCD cohort, childhood peer victimization (6–8 years) predicted depressive symptoms (15–17 years) whether the depression score was distributed continuously (beta = 0.256, SE = 0.075, *p*=<.001) or dichotomously (OR = 1.78, 95% confidence interval (CI): 1.20–2.61, *p* = .004). In the 1958BBC, peer victimization (7–11 years) predicted depressive symptoms at 50 years, according to both the continuous (beta = 0.151, SE = 0.066, *p* = .022) and dichotomous scores (OR = 1.35, 95% CI: 1.02–1.78, *p* = .033).

### Associations between the epigenetic indices and peer victimization or depressive symptoms

Childhood peer victimization was not associated with any of the epigenetic indices in both models in the QLSCD or the 1958BBC (Table 3). However, in both cohorts, significant associations were detected between epigenetic indices and depressive symptoms. In the QLSCD, PedBE derived from DNA collected at age 10 predicted higher depressive symptoms at age 17 (beta = .186, SE = .081, *p* = .023) in the unadjusted model, but this association was weakened when additional covariates sex, birthweight, and socioeconomic status were included in the model (beta = .139, SE = .078, *p* = .076) (Table 4). This association was, however, observed for the top 30% depressive symptoms in both adjusted and unadjusted models (OR = 1.65, 95% CI: 1.13–2.40, *p* = .010; OR = 1.55, 95% CI: 1.05–2.29, *p* = .026, respectively). Epigenetic pace of aging (DunedinPACE) was inversely associated with depressive symptoms, and a lower risk of reporting top 30% levels of depressive symptoms at the end of adolescence in this cohort (beta = −0.168, SE = .077, *p* = .030; OR = 0.65, 95% CI: 0.43–0.97, *p* = .034, respectively, in adjusted models).

**TABLE 3:** Linear regressions between epigenetic residuals and peer victimization.

QLSCD	Horvath1		Horvath2		PedBE		DunedinPACE		Epistress	
	Beta(SE)	*p*-value	Beta(SE)	*p*-value	Beta(SE)	*p*-value	Beta(SE)	*p*-value	Beta(SE)	*p*-value
Model 1										
Peer victimization 6-8y	0.012 (0.082)	0.887	0.000 (0.082)	0.997	−0.014 (0.082)	0.868	0.079 (0.082)	0.340	0.007 (0.082)	0.931
Model 2										
Peer victimization 6-8y	0.022 (0.084)	0.792	0.022 (0.082)	0.787	−0.004 (0.083)	0.960	0.079 (0.083)	0.345	0.012 (0.084)	0.888

1958BBC	Horvath1		Horvath2		PedBE		DunedinPACE		Epistress	
	Beta(SE)	*p*-value	Beta(SE)	*p*-value	Beta(SE)	*p*-value	Beta(SE)	*p*-value	Beta(SE)	*p*-value
Model 1										
Peer victimization 7-11 y	0.013 (0.065)	0.841	−0.077 (0.065)	0.236	0.024 (0.065)	0.710	0.057 (0.065)	0.379	0.045 (0.065)	0.485
Model 2										
Peer victimization 7-11 y	0.034 (0.067)	0.607	−0.090 (0.066)	0.176	0.017 (0.066)	0.803	0.057 (0.065)	0.385	0.059 (0.067)	0.378

Notes: Years (y). Epigenetic residuals adjusted principal components for cell type heterogeneity, smoking status at 42 years (1958BBC only), and body mass index (QLSCD: 10 years; 1958BBC: 45 years), and principal components for ancestry (QLSCD only), and chronological age (QLSCD: decimal age at the 10 years data collection; 1958BBC: decimal age at the 45 years data collection).

Max *N* based on data available for epigenetic clocks, DunedinPACE, and Epistress (QLSCD: *n* = 149; 1958BBC: *n* = 238). Model 1 was unadjusted; Model 2 is adjusted for sex, birth weight, and socioeconomic status (QLSCD: at 5 months; 1958BBC: at birth). QLSCD: Data were compiled from the final master file (1998–2015), © Gouvernement du Québec, Institut de la Statistique du Québec.

**TABLE 4:** Linear and logistical regressions between epigenetic residuals and depression.

	QLSCD				1958BBC			
	Depressive symptoms at 15-17 y				Depressive symptoms at 50 y			
	Continuous (z-scores)		Top 30% (dichotomized)		Continuous (z-scores)		Top 30%(dichotomized)	
	Beta(SE)	*p*-value	OR(95% CI)	*p*-value	Beta(SE)	*p*-value	OR(95% CI)	*p*-value
Model 1								
Horvath1	0.115 (0.082)	0.162	1.37 (1.14–1.65)	0.086	0.016 (0.066)	0.806	1.03 (0.89–1.19)	0.825
Model 2								
Horvath1	0.111 (0.078)	0.154	1.38 (1.14–1.67)	0.089	0.016 (0.066)	0.812	1.04 (0.89–1.20)	0.804
Model 1								
Horvath2	0.045 (0.082)	0.589	1.04 (0.86–1.24)	0.843	−0.071 (0.066)	0.287	0.90 (0.78–1.04)	0.465
Model 2								
Horvath2	−0.016 (0.079)	0.843	0.93 (0.78–1.12)	0.716	−0.056 (0.066)	0.402	0.91 (0.79–1.05)	0.517
Model 1								
PedBE	**0.186 (0.081)**	**0.023**	**1.65 (1.36**–**2.00)**	**0.010**	-	-	-	-
Model 2								
PedBE	0.139 (0.078)	0.076	**1.55 (1.27-1.89)**	**0.026**	-	-	-	-
Model 1								
DunedinPACE	−**0.190 (0.081)**	**0.020**	**0.65 (0.53-0.78)**	**0.024**	**0.147 (0.066)**	**0.028**	**1.34 (1.15**–**1.56)**	**0.047**
Model 2								
DunedinPACE	−**0.168 (0.077)**	**0.030**	**0.65 (0.53-0.79)**	**0.034**	0.124 (0.068)	0.071	1.32 (1.13-1.54)	0.068
Model 1								
Epistress	0.089 (0.082)	0.280	1.10 (0.92–1.32)	0.594	−0.005 (0.066)	0.941	0.97 (0.84–1.12)	0.825
Model 2								
Epistress	0.105 (0.078)	0.180	1.14 (0.94–1.37)	0.491	−0.009 (0.066)	0.894	0.97 (0.84–1.12)	0.821

Bold values are significant at *p* < 0.05. Notes: Years (y). Epigenetic residuals adjusted principal components for cell type heterogeneity, smoking status at 42 years (1958BBC only), and body mass index (QLSCD, 10 years; 1958BBC: 45 years), and principal components for ancestry (QLSCD only), and chronological age (QLSCD, decimal age at the 10 years data collection; 1958BBC: decimal age at the 45 years data collection). Max *N* based on data available for epigenetic clocks, DunedinPACE, and Epistress.

(QLSCD: *n* = 149; 1958BBC: *n* = 238). Model 1 was unadjusted; Model 2 is adjusted for sex, birthweight, and socioeconomic status (QLSCD: at 5 months; 1958BBC: at birth).

QLSCD, Data were compiled from the final master file (1998–2015), © Gouvernement du Québec, Institut de la Statistique du Québec.

In the 1958BBC, only the DunedinPACE positively predicted higher levels of depressive symptoms and a higher risk of top 30% depressive symptoms in unadjusted models (beta = −.147, SE = .066, *p* = .028; OR = 1.34, 95% CI: 1.00–1.80, *p* = .047, respectively). Epigenetic age estimates derived from the Horvath1 or Horvath2, and the Epistress score did not predict depressive symptoms in either the QLSCD or 1958BBC cohort.

### Associations between peer victimization, suicidal ideation, and epigenetic indices

In the QLSCD, each 1-SD increase in peer victimization at 6–8 years was associated to a 1.5 increased risk of suicidal ideation at 15–17 years in fully adjusted models which controlled for sex, socioeconomic status, and birthweight (OR = 1.57, 95% CI: 1.09–1.28, *p* = .016). In the 1958BBC, a 1-SD increase in peer victimization at 7–11 years was associated with a 1.7 increased risk of suicidal ideation at 45 years in the fully adjusted models (OR = 1.66, 95% CI: 1.03–2.66, *p* = 0.35). No association was detected between any of the epigenetic indices and suicidal ideation, in either cohort (Table 5).

**TABLE 5:** Logistical regressions between epigenetic residuals and suicidal ideation.

	QLSCD		1958BBC	
	Suicidal ideation at 15-17 years		Suicidal ideation at 45 years	
	OR (95% CI)	*p*-value	OR (95% CI)	*p*-value
Model 1				
Horvath1	0.94 (0.78–1.13)	0.727	0.87 (0.67–1.14)	0.611
Model 2				
Horvath1	0.92 (0.79–1.16)	0.836	0.88 (0.69–1.14)	0.634
Model 1				
Horvath2	1.11 (0.93–1.34)	0.555	0.72 (0.55–0.95)	0.232
Model 2				
Horvath2	1.09 (0.90–1.31)	0.658	0.76 (0.57–1.00)	0.317
Model 1				
PedBE	1.25 (1.03–1.50)	0.237	-	-
Model 2				
PedBE	1.19 (0.99–1.43)	0.355	-	-
Model 1				
DunedinPACE	0.96 (0.80–1.16)	0.835	1.48 (1.14–1.93)	0.133
Model 2				
DunedinPACE	1.01 (0.83–1.23)	0.954	1.29 (0.97–1.71)	0.361
Model 1				
Epistress	1.00 (0.83–1.21)	0.983	1.02 (0.78–1.33)	0.943
Model 2				
Epistress	1.04 (0.86–1.25)	0.851	1.03 (0.79–1.35)	0.904

Notes: Years (y). Epigenetic residuals adjusted principal components for cell type heterogeneity, smoking status at 42 years (1958BBC only), and body mass index (QLSCD: 10 years; 1958BBC: 45 years), and principal components for ancestry (QLSCD only), and chronological age (QLSCD, decimal age at the 10 years data collection; 1958BBC: decimal age at the 45 years data collection). Max N based on data available for epigenetic clocks, DunedinPACE, and Epistress (QLSCD: *n* = 149; 1958BBC: *n* = 238). Model 1 was unadjusted; Model 2 is adjusted for sex, birthweight, and socioeconomic status (QLSCD: at 5 months; 1958BBC: at birth). QLSCD: Data were compiled from the final master file (1998–2015), © Gouvernement du Québec, Institut de la Statistique du Québec.

Since no associations were found between the epigenetic indices and childhood peer victimization, as well as depressive symptoms or suicidal ideation, mediation analyses were not conducted.

## Discussion

This two-cohort study (i.e., QLSCD and 1958BBC) explored for the first time the role of three epigenetic indicators of aging (Horvath1, Horvath2 and PedBE), epigenetic pace of aging (DunedinPACE) and a cumulative epigenetic index capturing sensitivity to glucocorticoids (Epistress score) in association with childhood peer victimization, depressive symptoms, and suicidal ideation. The consistency of our results was showcased by three consistent patterns of findings uncovered in these two independent cohorts. First, childhood peer victimization predicted higher levels of depressive symptoms and risk of suicidal ideation in both adolescence (QLSCD) and adulthood (1958BBC). Second, childhood peer victimization was not associated with any of the epigenetic indicators of aging, pace of aging (DunedinPACE), or the Epistress score, at both 10 years (QLSCD) and 45 years (1958BBC). Third, none of the epigenetic aging indicators, the DunedinPACE, or the Epistress score predicted suicidal ideation in adolescence or adulthood. These similar observations are noteworthy because 1) they were tested in adolescence and adulthood, 2) they rely on prospectively collected data offering a stronger indication of the temporal sequence of events, 3) they emerged in samples composed of participants from distinct generations (participants born in 1997/1998 and 1958), who lived in different countries (Canada and the United Kingdom), and for whom different informants had reported peer victimization (self- and mother-reported experiences in the QLSCD and 1958BBC, respectively).

Non-etheless, some inconsistent findings were also detected, which may also point to other key differences between samples. Namely, the two cohorts used different tissues (blood in the 1958BBC; saliva in the QLSCD) collected in childhood vs. adulthood (45 years in the 1958BBC; 10 years in the QLSCD) to ascertain DNA methylation and derive the epigenetic indices. Furthermore, the PedBE was associated with depressive symptomology in the QLSCD, but we elected that the association could not be reliably tested in the 1958BBC due to concerns over the validity of the epigenetic age estimate predicting the chronological age, and because it did not covary with another epigenetic indicator of aging. Furthermore, this index was originally derived in saliva samples and younger samples (less than 20 years of age). Our findings thus contrasted with the PedBE shown to correlate with chronological age in youth using blood samples, as well as in adult saliva samples (McEwen et al., 2020). Our PedBE estimates suggest that it is not optimized for blood samples later in life. It is also important to note that the Epistress score has been validated in blood samples, but not in saliva samples (Provençal et al., 2020). Thus, further studies would be needed to understand whether this index is suitable for use in saliva samples and beyond the perinatal period. That is, it is important to account for differences between cohorts (age and tissue-type) as putative factors underlying inconsistent results between the cohorts.

### Associations between epigenetic indices and peer victimization

We did not detect associations between the Horvath1 (10 years) and our averaged self-reported measures of peer victimization (6–8 years), which is consistent with the finding reported by the only prior study that had tested the association between bullying experienced before the age of 14 and this index measured from DNA collected at 17 years (Tang et al., 2020). Our study extended this search into adulthood (1958BBC), and according to other indicators of epigenetic aging (PedBE in the QLSCD, and Horvath2 in both cohorts). These null findings are in line with some studies focusing more generally on early childhood adversity, although the evidence is also inconsistent overall. For example, while some studies did not observe associations between cumulative adversity and epigenetic age estimates (Wolf et al., 2018; Marini et al., 2020; Hamlat et al., 2021), others have reported significant associations with the epigenetic aging indicators (Sumner et al., 2019; Tang et al., 2020). The epigenetic indicator of pace of aging (DunedinPACE) was not associated with peer victimization in the QLSCD or the 1958. Prior work had linked faster epigenetic pace of aging with polyvictimization (Bourassa et al., 2021), however it is possible that peer victimization alone does not predict faster aging.

Our inclusion of the Epistress score known to be sensitive to stress, also failed to uncover any new evidence for an association with peer victimization. More generally, this study reminds us of the difficulty of replicating earlier findings related to peer victimization on DNA methylation differences in specific CpG sites in the *SERT* and *NR3C1* genes (Ouellet-Morin et al., 2013; Efstathopoulos et al., 2018) or dispersed across the genome (Marzi et al., 2018; Mulder et al., 2020). Such negative findings remain important as they contribute to expand on this scarce literature and reflect on the complexity of identifying the molecular pathways transducing social adversity to poorer later health. Future studies with greater statistical power could also investigate these associations into their biological (genes, other epigenetic biomarkers), psychological (e.g., emotion regulation, coping strategies) and social contexts (e.g., social support, social norms discouraging violence).

### Associations between epigenetic indices, depressive symptoms, and suicidal ideation

The Horvath1 was not associated with depressive symptoms in adolescence or adulthood. This finding is somewhat inconsistent with the previous cross-sectional and longitudinal associations reported between this epigenetic aging indicator derived from DNA collected at 6 years and internalizing symptoms at ages 6, 7, and 10 years (Tollenaar et al., 2021). However, there are differences in study design that could explain the apparent inconsistency. First, internalizing problems include depression as well as anxiety symptomatology, thus it is unclear whether the association with Horvath1 would be linked to the comorbid presence of both anxiety and depression symptomatology or their severity. Secondly, symptoms were measured in childhood while we measured depression in mid to late adolescence when these symptoms are more common (Maughan et al., 2013). This difference in the timing of symptom assessment may have contributed to differences across studies. Of note, although our association were not significant, Horvath1 was nevertheless associated with a risk of reporting top 30% depressive symptoms scores at trend level in the QLSCD (*p* = 0.08). Lastly, one study found that the Horvath1 was cross-sectionally associated with depressive symptoms in youth aged between 8–16 years (Sumner et al., 2019), using similar control variables to our study. Our study however was longitudinal rather than cross-sectional since epigenetic aging estimates were calculated from DNA methylation measured several years before symptoms. It is possible that cross-sectional study captured transient, concurrent effects that lessen over time. More studies with similar designs will help compare and further understand differential findings. Alternatively, we did find that the PedBE predicted elevated depressive symptoms in adolescence (QLSCD). Our finding is nevertheless inconsistent with a prior study reporting that PedBE at 6 years was not associated with internalizing problems from 6 to 10 years in a community sample (McGill et al., 2022). The presence of a significant association between depressive symptoms and the PedBE, but not the Horvath1 scores, may capture biological mechanisms involved with aging that may be differentially related with depression, in term of subtypes, severity, or persistence. Future studies could further investigate this possibility. Furthermore, DunedinPACE predicted fewer depressive symptoms in adolescence (QLSCD), after accounting for childhood socioeconomic status. The same epigenetic indicator of pace of aging was associated to socioeconomic disadvantage in youth (Raffington et al., 2021) and adulthood (McCrory et al., 2022). We speculate that a slower pace of aging may reflect a delayed development in childhood (or other accounted individual characteristics) which may in turn heighten the risk to develop depressive symptoms in adolescence. In adulthood, a faster pace of aging was found with depressive symptoms. However, this association weakened after controlling for sex and socioeconomic status to trend level (*p* = 0.07).

For the first time, this study tested the association between the Epistress score and depressive symptoms. Although the Epistress is a novel score, it was thought to be a suitable potential mediator due to prior knowledge on HPA axis reactivity in association to depressive symptoms, and early life stress. However, in our study, the Epistress scores were not associated with neither depression nor peer victimization. Provençal et al. (2020) had reported that lower Epistress scores in newborn cord blood were associated with higher prenatal maternal depression. Associations between the Epistress and depression in individual participants has not been tested before. Overall, previous literature on HPA axis reactivity and depression remains inconsistent, likely due to variations in study design (Hammen, 2015). Further studies are needed to clarify if the Epistress score and depression (or peer victimization) association is more likely to occur at specific periods of life (e.g., perinatal, vs. childhood).

Neither epigenetic indicators of aging or pace of aging, nor Epistress score, predicted suicidal ideation in either cohort. Dada et al. (2020) did not find a cross-sectional association between Horvath1 and suicidal ideation in patients with schizophrenia. It is important to note, however, that our measure of suicidal ideation captured a broad phenotype of suicidal ideation which did not specifically capture suicidal ideation as well as behaviors (e.g., attempts); with passive suicidal ideation (without information on plan or intent) in the QLSCD, and depressive ideas (including hopelessness, worthlessness, and thoughts of death) in the 1958BBC. A recent review found that most studies have reported an association between epigenetic changes and suicide attempts, but not ideation (Dada et al., 2021), which may partly explain the lack of association in the present study. Considering the scarcity of studies that examined this association, and the somewhat small size of the cohorts used in the current analysis, it would be premature to dismiss a possible association between epigenetic aging and suicidal ideation.

### Study limitations

While this study has many strengths, including the two-cohort study design, there are important limitations to take into consideration. First, information about DNA methylation was available only in subsamples of these cohorts, which precludes generalization to the larger population, and which may have constrained our power to detect associations small effect sizes. Furthermore, some differences between our study subsamples and the larger cohorts were noted which calls for caution in the generalization of our finding to the general population. Second, DNA was extracted from peripheral tissues which may not reflect mechanisms in the central nervous system involved in the stress response or the onset of depressive symptoms or suicidal ideation. One future avenue to account for the use of peripheral most accessible tissues (e.g., blood, saliva) may be to compute indicators of aging which include CpG sites that are conserved from DNA derived from brain tissues to blood and saliva samples (Grodstein et al., 2021). Third, we did not have information on pubertal timing at 10 years in the QLSCD, which warrants attention since early pubertal onset is linked to epigenetic aging (Hamlat et al., 2021) and pace of aging (Raffington et al., 2021). Fourth, peer victimization was self-reported using six items in the QLSCD, while it was measured using a single mother-reported item in the 1958BBC. The use of different raters has been supported by prior studies showing that although mother-reports and self-reports are moderately correlated, they both associated similarly with health outcomes (Shakoor et al., 2011). Additionally, it is possible that our measures of peer victimization did not capture frequent and repeated peer victimization as we averaged scores over several time points. Future studies could investigate whether more severe and persistent experiences of peer victimization relate to cumulative epigenetic indices. Fifth, we used an arbitrary cut-off to identify elevated depressive symptoms (top 30% scores). Even though this cut-off score is not synonymous with clinical depressive symptom levels, it provided an opportunity to explore the possibility that DNA methylation markers may have had a distinct association with depressive symptoms at the higher end of the distribution, and to apply this strategy similarly in our two cohorts. Lastly, while indicators of epigenetic aging, pace of aging, and Epistress may not relate to peer victimization, it is possible that other epigenetic biomarkers and mechanisms may be involved.

## Conclusion

To conclude, no associations were found between peer victimization, suicidal ideation, and the epigenetic indices. Inconsistent findings have been detected between the epigenetic indices and depression. Perhaps one indicator of epigenetic aging alone does not reflect the full complexity of biological aging on a molecular level. One possibility could be to adopt a composite epigenetic indicator approach, as suggested by Jansen et al. (2021), to account for several aging indicators that could cumulatively explain a greater portion of variance than independent CpGs and aging indicators. As research on the association between early adverse social experiences and epigenetic aging is at its infancy further studies are needed to advance our understanding of the biological mechanisms behind adversity and to directly test whether changes in DNA methylation relate to depression and suicidality.

## Data Availability

The data analyzed in this study is subject to the following licenses/restrictions: The data analyzed in this study was obtained from the Institut de la Statistique du Québec and, as stipulated in the clauses 10 and 11 of the Institut de la statistique’s Québec Act (Canada), the access to the data is restricted to the parties identified in the partnership agreement signed to ensure the conduct of the study and which describes the author’s right. In the QLSCD cohort, the participants only consented to share their data to the study’s financial partners and affiliated researchers and their collaborators. Those partners and researchers have only access to the data after signing a data sharing agreement. Requests to access these data can be directed to the Institut de la Statistique du Québec’s Research Data Access Services—Home (https://statistique.quebec.ca/en). For more information, contact SAD@stat.gouv.qc.ca. The pseudonymized individual-level genetics data from the 1958BBC cohort analyzed in this study were obtained from the Centre for Longitudinal Studies (CLS), based at University College London, which is the Data Controller as defined by the United Kingdom General Data Protection Regulation (United Kingdom GDPR), tailored by the Data Protection Act 2018. To protect the fundamental legal rights and freedoms of these individuals and, in particular, their right to privacy, access to these data is solely governed by the CLS Data Access Committee (CLS DAC). Researchers can have access to these data following the application to and approval of their project by the CLS DAC, with data release being subject to signing the CLS data sharing agreement. Information about how to access these data can be found at https://cls.ucl.ac.uk/data-access-training/genetic-data-and-biological-samples/. For more information, contact the CLS Data User Support at clsfeedback@ucl.ac.uk.

## Supplementary material

Supplementary Figure S1 Two-cohort study design with variables of interest and main confounders. Note: y, years; mo, months; mDNA, DNA methylation; BMI, Body Mass Index; PCs, Principal Components.
