# Supplementary figures and images for: Associations between epigenetic aging and childhood peer victimization, depression, and suicidal ideation in adolescence and adulthood: A study of two population-based samples
Source: Front Cell Dev Biol. 2023 Jan 12;10:1051556. doi: 10.3389/fcell.2022.1051556 (PMC9879289; doi:10.3389/fcell.2022.1051556)

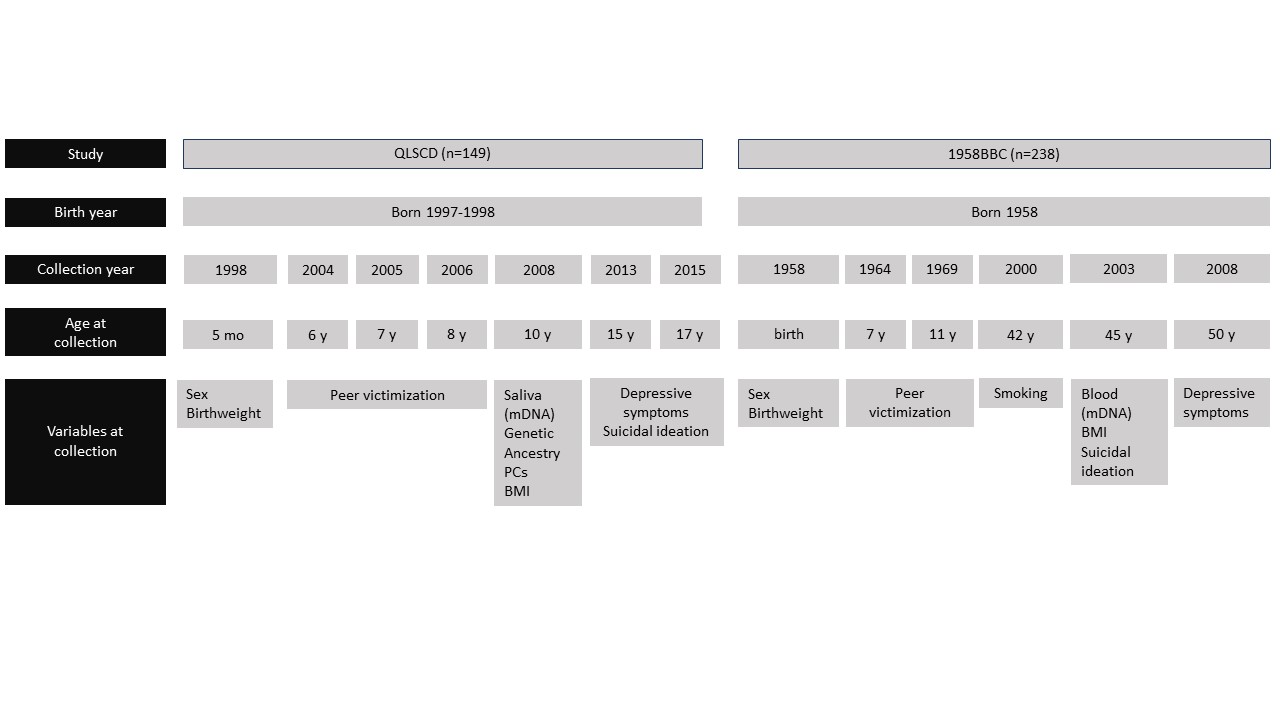

Supplement: Supplementary file 1 [file Image1.jpg]
